# A case of basidiobolomycosis mimicking rhabdomyosarcoma: A diagnostic challenge

**DOI:** 10.1016/j.radcr.2022.07.004

**Published:** 2022-07-22

**Authors:** Rasha Alskaff, Anas Alkhudari, Fawaz Skaff, Belal Nedal Sabbah, Moheieldin M. Abouzied

**Affiliations:** a College of Medicine, Alfaisal University, Riyadh, Saudi Arabia; b Department of Radiology, Medical Imaging Services, King Faisal Specialist Hospital and Research Center, Riyadh, Saudi Arabia

**Keywords:** Basidiobolomycosis, Radiology, Rhabdomyosarcoma

## Abstract

Basidiobolomycosis is a rare curable fungal infection caused by the saprophytic fungus Basidiobolus ranarum. It often causes skin infections but rarely infects visceral tissues in humans. Gastrointestinal basidiobolomycosis is an emerging form, which is rare but is increasingly reported. Due to its ability to mimic more common diagnoses such as chronic inflammatory disorders and malignancies, Basidiobolomycosis imposes a diagnostic challenge on most physicians. Therefore, a timely and correct diagnosis by laboratory tests and careful review of images along with proper medical management can save patients from invasive treatments and reduce both morbidity and mortality. Here, we present a rare case of an 8-year-old boy with basidiobolomycosis initially misdiagnosed as rhabdomyosarcoma. We aim to highlight basidiobolomycosis as a potential differential from masses on imaging under the right clinical circumstances and to provide radiologists with key imaging details to help recognize this infectious etiology and reduce its associated morbidity.

## Introduction

*Basidiobolus ranarum* is an environmental saprophytic fungus found in dead plant material, rotten wood, soil, reptile feces, insectivorous fish, and amphibians, and is mainly limited to tropical regions; the Middle East, Africa, and South America [1]. It belongs to the order Entomophthorales of the class Zygomycetes. Basidiobolomycosis is characterized by chronic subcutaneous induration affecting the limbs, trunk, and buttocks. Minor trauma, local inoculation, and insect bites appear to be the predominant modes of acquisition [2]. Although visceral involvement is rare, it appears to be an emerging fungal infection in Saudi Arabia, Iran, Iraq, and Arizona in the United States of America [3].

Extracutaneous manifestations are diverse and nonspecific depending on the tissue involved; patients with B. ranarum infection may present with subcutaneous, gastrointestinal, or systemic lesions [4]. Consequently, gastrointestinal basidiobolomycosis (GIB) is recognized in the literature as a cause of misdiagnosis due to its ability to mimic the manifestations of more common entities such as benign tumors, malignancies, inflammatory bowel disease, appendicitis, tuberculosis, etc. [2].

Due to the varied presentations associated, several diagnostic modalities have been used to identify the cause. Medical imaging has been commonly used to identify the cause of symptoms in patients with basidiobolomycosis who had not yet received this curable diagnosis; In a recent study with a moderate sample size of 30 patients, 68% have performed computed tomography in their study [2].

In hope of better identification of basidiobolomycosis with the aim of reducing associated morbidity and mortality [1], we here present a case in which the infection was initially misdiagnosed as rhabdomyosarcoma, with an emphasis on its radiological findings that have raised suspicion of an infectious cause rather than malignancy later on. Upon review of the literature, only 7 similar cases have been reported between 2012 and 2022, some of which have stated rhabdomyosarcoma as an initial differential to the mass formed by Basidiobolomycosis [5]. We aim to highlight basidiobolomycosis as a potential differential to masses on imaging under the right clinical circumstances [6], so we can provide the appropriate therapy as soon as possible, as the duration of morbidity of this infection can last months [2].

## Case presentation

An 8-year-old male patient from the southern province of Saudi Arabia presented to a local hospital with perianal pain, constipation, and gluteal swelling for 3 months. He also reported intermittent low-grade fever. He was diagnosed with gluteal abscess and underwent an incision only. He was discharged on antibiotic; however, no improvement was noticed. Pelvic magnetic resonance imaging (MRI) was performed and bladder and gluteal masses were found. He was referred to our hospital as a potential case of rhabdomyosarcoma. On arrival, he complained of on and off fever, decreased oral intake, dysuria, and frequency with a small amount of urine. He also complained of back pain that was partially relieved by acetaminophen. Physical examination showed an ill, febrile and underweight child with a firm erythematous gluteal mass extending to the peri-anal area with bilateral inguinal lymphadenopathy.

Laboratory investigations showed hemoglobin (10.6 g/dl) [Normal range (10.2-15.2 g/dL)], hematocrit (0.353 L/L) [Normal range (0.35-0.40 L/L)], mean corpuscular volume (59 fL) [Normal range (74-91 fL)], white blood cell count (16.74 × 10^9^/L) [Normal range (4.30-11.30 × 10^9^/L)], absolute neutrophils (71.5 × 10^9^ cells/L) [Normal range (1.35-7.50 × 109 cells/L)], lymphocytes (7.8 × 10^9^ cells/L) [Normal range (1.5-7.0 × 10^9^ cells/L)], monocytes (5.8 × 10^9^ cells/L) [Normal range (0.2-1 × 10^9^ cells/L)], absolute eosinophils (2.3 × 10^9^ cells/L) [Normal range (0.03-1 × 10^9^ cells/L)], basophils (0.4 × 10^9^ cells/L) [Normal range (0-0.2 × 10^9^ cells/L)], ESR (88mm / H) [Normal range (0-36 mm/ H)] and CRP (205 mg/dl) [Normal range (0.8-1.0 mg/dL)] and creatinine (44 mg/dl) [Normal range (0.5-1.0 mg/dL)].

**Fig. 1:**
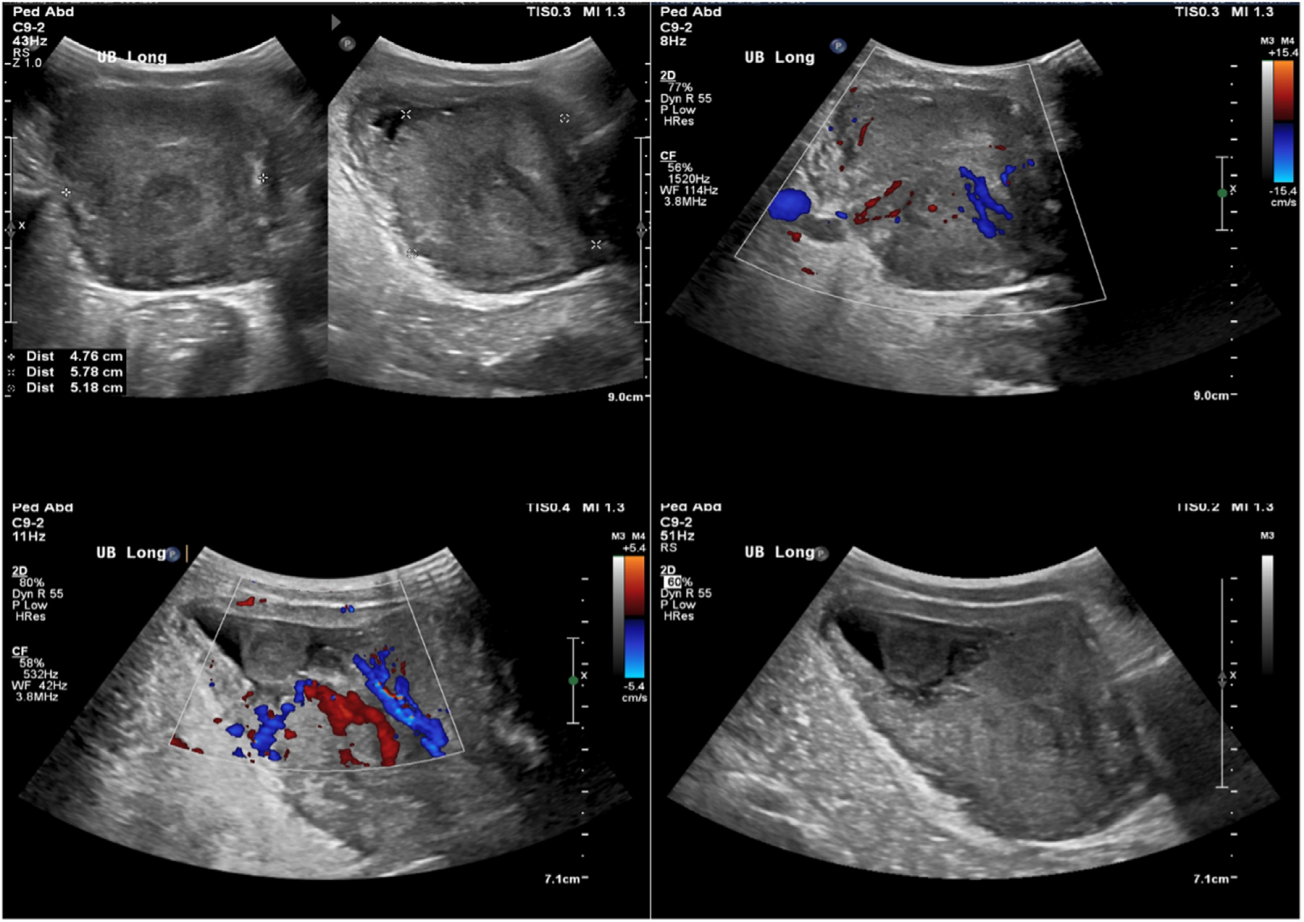
Pelvic ultrasound showed hypervascular large mass with difficulty to define its origin, either from bladder or prostate.

**Fig. 2:**
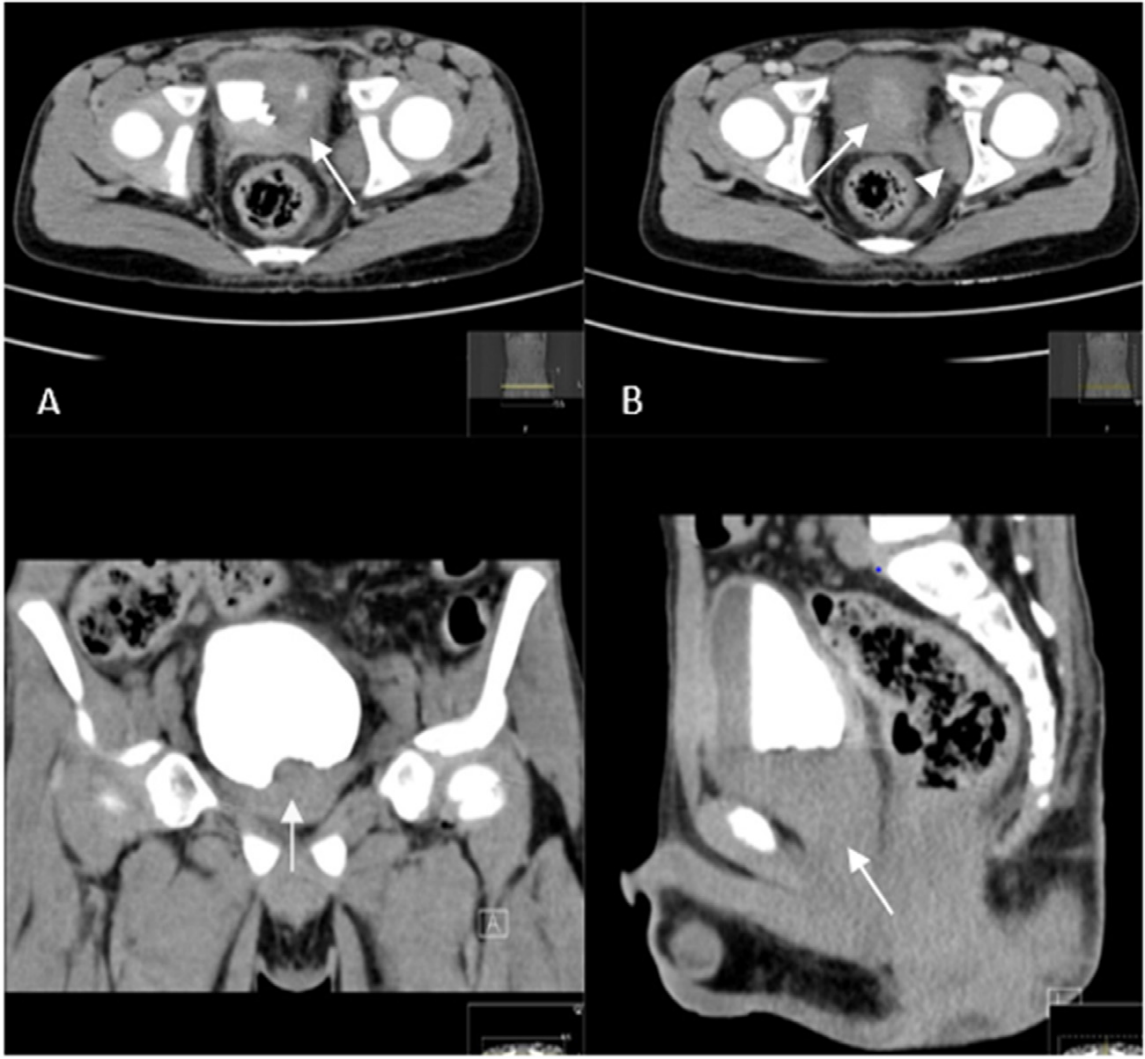
Axial section of delayed images CT scan showed (A) contrast filled bladder with partial filling defect at the level of bladder's trigone. (White arrow), and (B) mild hyperdense deep pelvic mass lesion inseparable from the prostate (white arrow) and enlarged left internal obturator muscle. (Arrow head). (C & D) Coronal and sagittal cut images showed deep pelvic mass lesion without distinct origin either from bladder or prostate, respectively. (White arrow).

**Fig. 3:**
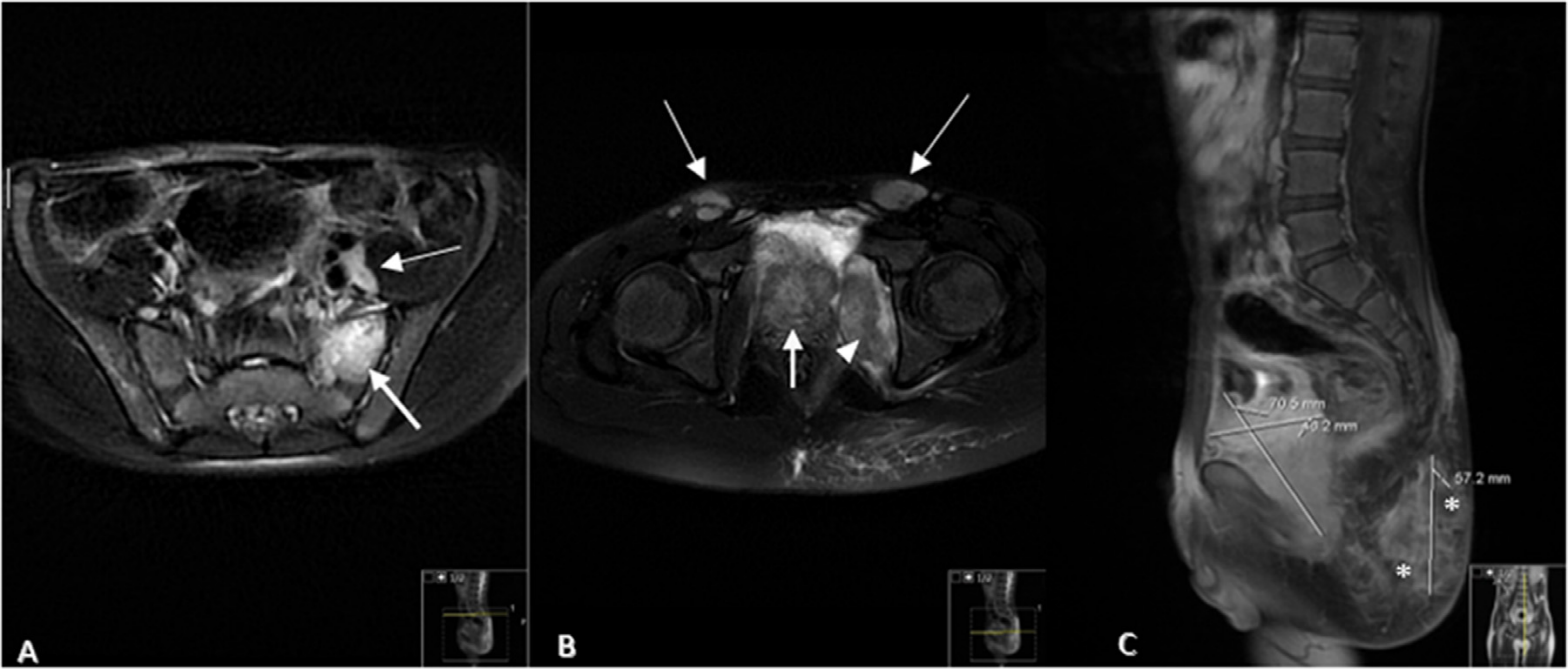
Axial T2- FS image showed (A) high signal intensity in the left sacral ala (white arrow) and left external iliac lymph node enlargement (thin white arrow). And (B) showed heterogeneous increased signal intensity of pelvic mass (white arrow), swelling of left internal obturator muscle (arrow head) and bilateral inguinal lymph nodes enlargement (thin white arrows). (C) Sagittal T1- FS contrast image showed enhancement of the pelvic mass, heterogeneous enhancement of the gluteal lesion with central hypo-intensity suggesting necrosis/collection (asterisk).

**Fig. 4:**
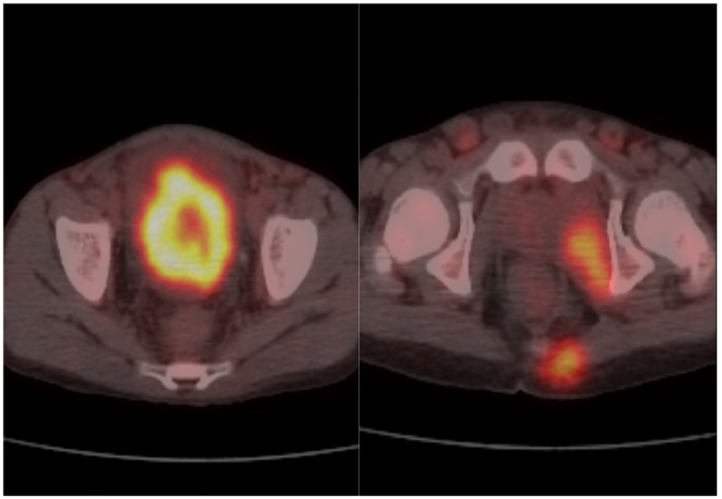
Axial 18F-FDG PET CT scan fused image showed (A) hypermetabolic pelvic mass, and (B) hypermetabolic left internal obturator muscle, partially visualized left gluteal lesion and mildly hypermetabolic bilateral inguinal lymph nodes.

Blood and urine cultures were negative. A series of images including ultrasound, CT scan, MRI and PET/CT scan were performed and a mass arising from the bladder base/prostate with intravesical extension likely representing bladder base rhabdomyosarcoma with tumor extension to the left pelvic sidewall and inversion to the internus muscle of the left obturator. Additionally, a large left gluteal subcutaneous mass and bilateral enlarged inguinal lymph nodes suggested metastasis (Fig. 1, Fig. 2, Fig. 3, Fig. 4) Ultrasound showed echogenic kidneys with left hydroureteronephrosis.

An initial diagnosis of rhabdomyosarcoma and acute kidney injury secondary to obstructive uropathy and contrast-induced injury was made. Accordingly, the patient was scheduled for biopsy, Port A Cath insertion, and bilateral nephrostomy tube insertion. He was started on empirical antibiotics for the fever and high white blood counts. Cystoscopy guided bladder neck and posterior urethral biopsies showed benign urothelium with polypoid cystitis and acute and chronic inflammations. The biopsies were probably not representative and inconsistent with the radiologically described possible rhabdomyosarcoma. Therefore, the patient was referred for lymphoma workup and scheduled for bone marrow biopsy and repeated cystoscopy-guided biopsy. The bone marrow biopsy result showed normocellular bone marrow with 90% cellularity without morphological evidence suggesting leukemia, lymphoma, or nonhematopoietic malignancies.

**Fig. 5:**
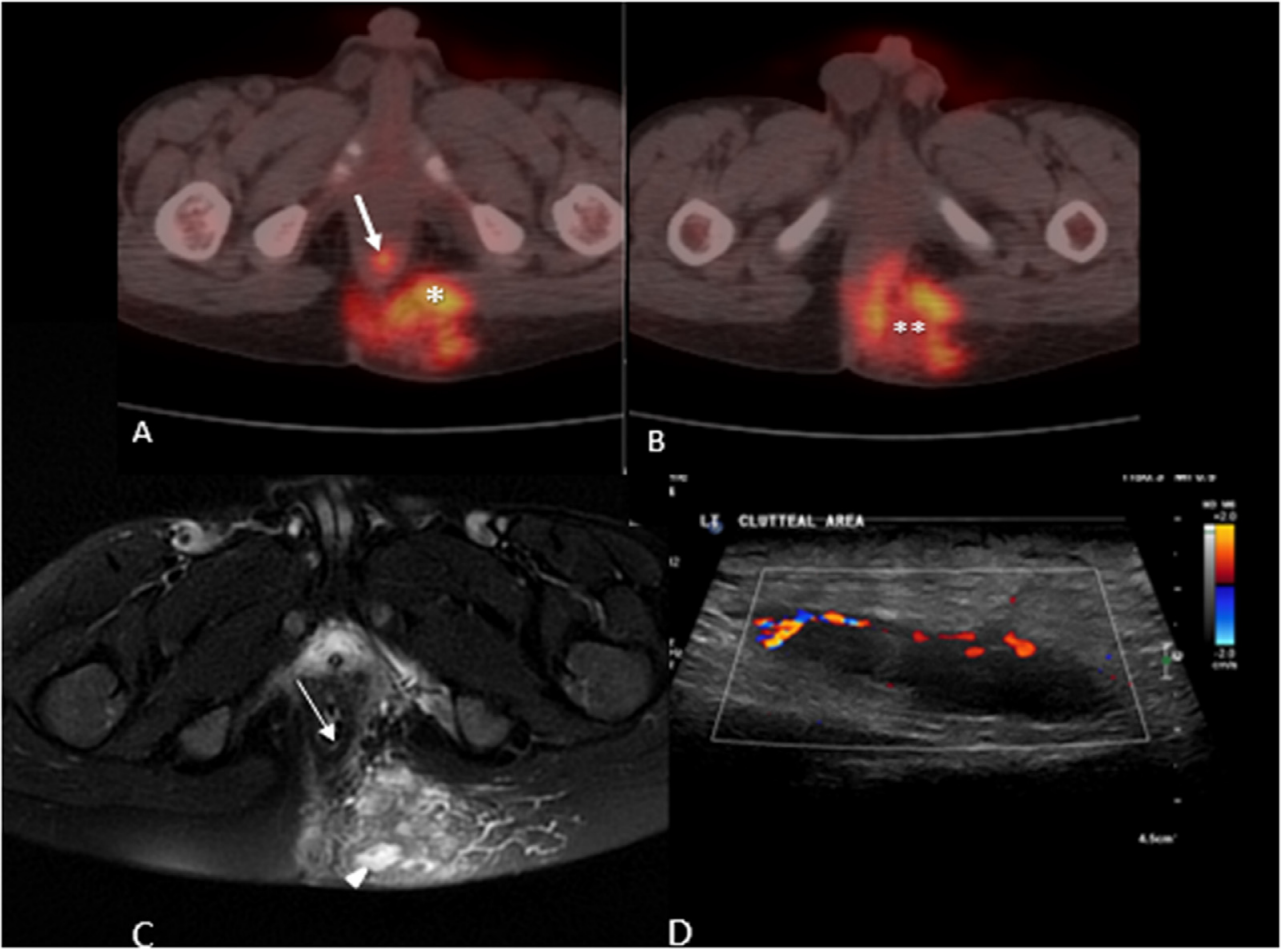
(A) 18 F-FDG PET/CT scan showing intersphincteric focal hypermetabolism suggestive of fistulous tract (White arrow) and hypermetabolism of left gluteal lesion (asterisk). (B) 18 F-FDG PET/CT scan showed mild central photopenia of the left gluteal lesion suggestive of necrosis/collection. (Double asterisks). (C) Axial MRI T2-FS showing heterogeneous signal intensity of the left gluteal lesion with focal high signal intensity suggestive of necrosis/collection (arrow head) and faintly visualized intersphicteric increased signal intensity suggestive of fistulous tract raising the suspicion of infectious/inflammatory process. (Thin white arrow). (D) Gluteal ultrasound showing anechoic structure with peripheral hypervascularity suggesting collection.

Upon a second review of the images by different radiologists to delineate the nature and source of the mass by analyzing and comparing different imaging modalities, a suspicion of infectious/inflammatory cause was raised due to several findings. The presence of the intersphincteric fistulous tract associated with focal left gluteal collection as shown on PET / CT, MRI, and ultrasound (Fig. 5). As a result, the diagnosis of malignancy and necrosis was less likely.

**Fig. 6:**
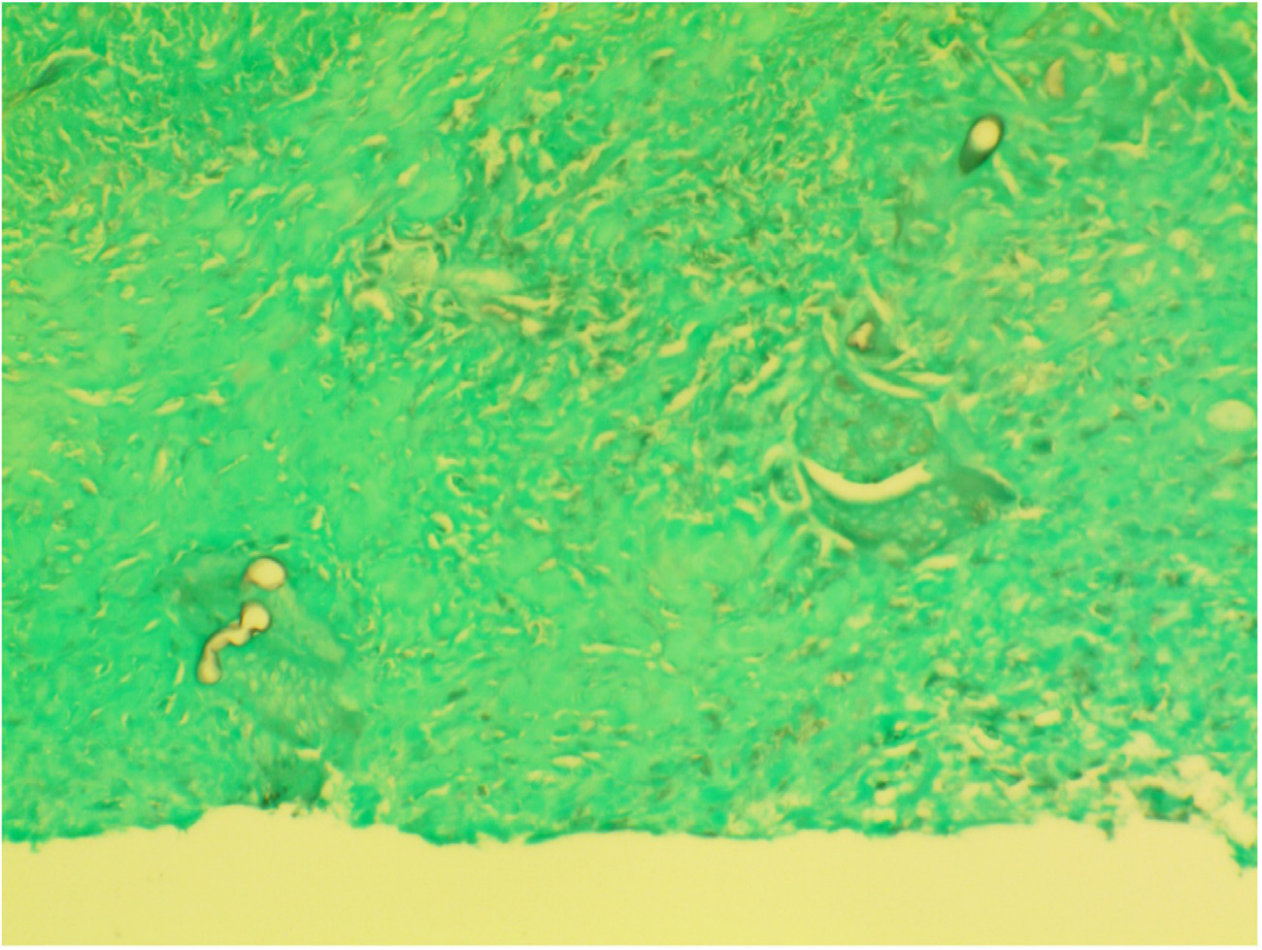
Grocott's methenamine silver stain showing fungal hyphae and spores (arrows) confirming Basidiobolomycosis diagnosis.

**Fig. 7:**
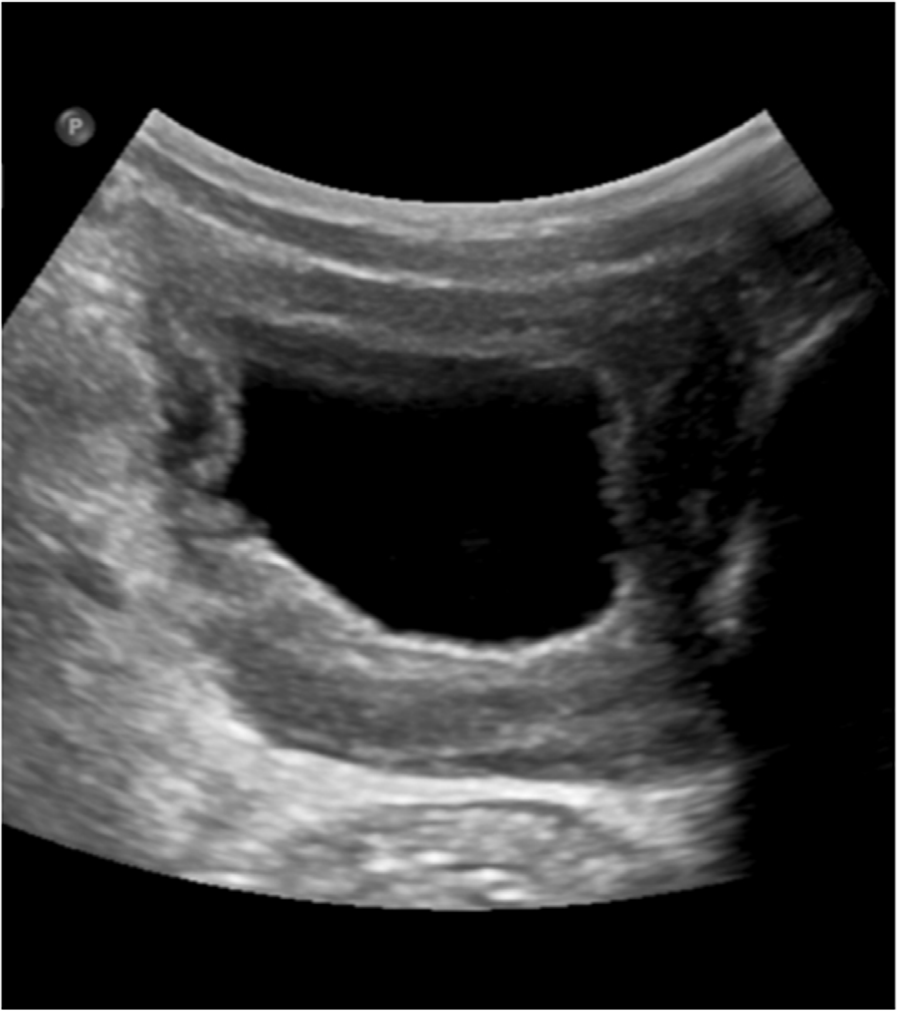
Follow up ultrasound of the bladder showing complete resolution of the pelvic mass.

Radiologist recommended a pelvic ultrasound-guided biopsy and the results showed mixed acute and chronic inflammation with multinucleate giant cells and prominent eosinophils. Periodic acid-Schiff (PAS) and Grocott methenamine silver (GMS) stains highlight hyphal fungal organisms suggesting basidiobolomycosis (Fig. 6). The patient was started on a prolonged course of Voriconazole with excellent response. Laboratory investigations showed a marked improvement in the patient's blood and kidney functions. Complete resolution of the bladder and gluteal masses was noted in ultrasound follow-up (Fig. 7), which saved him from invasive surgical resection of the mass.

## Discussion

Basidiobolomycosis is a rare emerging fungal infection caused by Basidiobolus ranarum. In the literature, more than 102 cases of GIB published between 1997 and 2018 and reviewed by Mohammadi et al. [1]. It belongs to the Zygomycetes class, which includes 2 fungal orders: Mucorales and Entomophthorales. The Mucorales include Mucor species and Entomophthorales include B. ranarum, which infect immunocompromised and immunocompetent individuals, respectively [7], [8], [9]. Most reported cases come from arid regions of the world, including Arizona in the United States, Iran, Iraq, Saudi Arabia, and Kuwait [7]. It is generally found in a temperate and humid environment mainly in soil and decaying vegetables, and also in the intestines of some animals such as fish, dogs, and amphibians [2,10].

B. ranarum infects both adult and pediatric populations with male predominance, while the majority of pediatric cases were reported from Saudi Arabia [10]. Basidiolobolomycosis commonly causes a characteristic subcutaneous lesion mainly in the trunk, buttocks, or extremities [11]. It rarely causes visceral lesions; however, gastrointestinal and pelvic involvement has become an emerging manifestation of B. ranarum infection. The involvement of primary gastrointestinal sites includes the colon, stomach, and small intestine [12]. Common presentation of basidiobolomycosis includes fever, abdominal pain, constipation, and abdominal mass. Many factors contribute to the difficulty in diagnosing GIB, including nonspecific clinical presentation, the absence of predisposing risk factors, and suboptimal biopsy results. Therefore, it is often misdiagnosed as malignancy, intestinal tuberculosis, or inflammatory bowel disease.

Most reported cases showed masses located in the colon, liver, small bowel, or multiple locations, and concentric bowel wall thickening on abdominal imaging, mainly CT. Therefore, the differential diagnosis of neoplasms and IBD was initially suggested [6]. Additionally, some cases reported abscess formation implying a potential infectious cause such as tuberculosis. Meanwhile, our case showed a large pelvic mass that was difficult to identify its origin with a subcutaneous gluteal lesion that was first mistaken for rhabdomyosarcoma. It is important to remember that rhabdomyosarcoma is the most common pelvic soft tissue sarcoma in children. It usually arises from the genitourinary system and commonly affects the bladder [13]. Generally, MRI findings of rhabdomyosarcoma often show hypointense to isointense on noncontrast T1-weighted images and isointense to hyperintense on T2-weighted sequences. They frequently have areas of hyperintensity without enhancement representing necrosis on water sensitive sequences [14]. Furthermore, regional and distant lymph node metastases may be present that mimic lymphoma or epithelial malignancies [14]. However, it is less likely to present with an intersphincteric fistula, as seen in our case. It is worth mentioning the trigger findings that suggested an infectious cause rather than rhabdomyosarcoma in our case, as an intersphincteric fistulous tract associated with focal left gluteal collection was seen.

A high index of suspicion of GIB should be maintained in the differential diagnosis of patients with abdominal mass, subcutaneous lesion, and eosinophilia along with a negative biopsy for neoplasms. In addition to findings suggestive of infectious/inflammatory changes such as fistulas and collections on radiographic images. The gold standard diagnosis of basidiobolomycosis is the histopathologic evaluation of the biopsy [1]. The treatment modality mentioned in the literature ranged between medical and surgical intervention [15,16]. It is important to state that the prolonged course of antifungal medication from the -azole family showed complete resolution of infection [15,17] follow-ups without surgical interventions.

We emphasize the importance of early recognition of this rare fungal infection by carefully evaluating different imaging modalities. Thus, preventing patients from undergoing unnecessary surgical intervention and reducing complications and morbidity.

## Provenance and peer review

Not commissioned, externally peer-reviewed

## Ethical approval

Patient anonymity is maintained throughout this manuscript, and consent was obtained for publication from the patient

## Author contribution

R.A., A.A., F.S., B.N.S., M.M.A. drafted the manuscript B.N.S contributed to reviewing and finalizing the manuscript. F.S. provided the imaging findings and their interpretation for the case presentation section. All authors reviewed the manuscript for intellectual content and approved the submission.

## Consent for publication

Written informed consent was obtained from the patient's family for publication of this case report and accompanying images. A copy of the written consent is available for review by the Editor-in-Chief of this journal on request.

## References

[1] Mohammadi R, Ansari Chaharsoghi M, Khorvash F, Kaleidari B, Sanei MH, Ahangarkani F (2019). An unusual case of gastrointestinal basidiobolomycosis mimicking colon cancer; literature and review. J Mycol Med.

[2] Vikram HR, Smilack JD, Leighton JA, Crowell MD, de Petris G. (2012). Emergence of gastrointestinal basidiobolomycosis in the United States, with a review of worldwide cases. Clin Infect Dis.

[3] Pezzani MD, di Cristo V, Parravicini C, Sonzogni A, Tonello C, Franzetti M (2019). Gastrointestinal basidiobolomycosis: an emerging mycosis difficult to diagnose but curable. Case report and review of the literature. Travel Med Infect Dis.

[4] Zekavat OR, Abdolkarimi B, Pouladfar G, Fathpour G, Mokhtari M, Shakibazad N. (2017). Colonic basidiobolomycosis with liver involvement masquerading as gastrointestinal lymphoma: a case report and literature review. Rev Soc Bras Med Trop.

[5] Almoosa Z, Alsuhaibani M, AlDandan S, Alshahrani D. (2017). Pediatric gastrointestinal basidiobolomycosis mimicking malignancy. Med Mycol Case Rep.

[6] Flicek KT, Vikram HR, de Petris GD, Johnson CD. (2015). Abdominal imaging findings in gastrointestinal basidiobolomycosis. Abdom Imaging.

[7] A child with intestinal basidiobolomycosis n.d. Available at: https://www.ncbi.nlm.nih.gov/pmc/articles/PMC3470076/(Accessed June 18, 2022).

[8] Mohammed SA, Abdelsatir AA, Abdellatif M, Suliman SH, Elbasheer OMI, Abdalla AR (2020). Challenging presentations of seven cases of gastrointestinal basidiobolomycosis in Sudan: clinical features, histology, imaging, and recommendations. J Lab Physicians.

[9] Gastrointestinal basidiobolomycosis, a rare and under-diagnosed fungal infection in immunocompetent hosts: a review article - PubMed n.d. Available at: https://pubmed.ncbi.nlm.nih.gov/25821287/(Accessed June 18, 2022). PMC4359942 25821287

[10] El-Shabrawi HFM, Kamal NM, Jouini R, Al-Harbi A, Voigt K, Al-Malki T (2011). Gastrointestinal basidiobolomycosis: an emerging fungal infection causing bowel perforation in a child. J Med Microbiol.

[11] Humber RA. Entomophthoromycota: a new phylum and reclassification for entomophthoroid fungi Mycotaxon 2012;120:477–92. doi:10.5248/120.477.

[12] Pasha TM, Leighton JA, Smilack JD, Heppell J, Colby T v., Kaufman L (1997). Basidiobolomycosis: an unusual fungal infection mimicking inflammatory bowel disease. Gastroenterology.

[13] Skapek SX, Ferrari A, Gupta AA, Lupo PJ, Butler E, Shipley J (2019). Rhabdomyosarcoma. Nat Rev Dis Primers.

[14] Saboo SS, Krajewski KM, Zukotynski K, Howard S, Jagannathan JP, Hornick JL (2012). Imaging features of primary and secondary adult rhabdomyosarcoma. AJR Am J Roentgenol.

[15] al Haq AM, Rasheedi A, Farsi M, Mehdar A, Yousef Y, Rasheed K (2021). Gastrointestinal Basidiobolomycosis in pediatric patients: a diagnostic dilemma and management challenge. Int J Pediatr Adolesc Med.

[16] Rabie ME, Qahtani AS, Jamil S, Mikhail NT, Hakeem I, Hummadi A (2019). Gastrointestinal basidiobolomycosis: an emerging potentially lethal fungal infection. Saudi Surg J.

[17] Geramizadeh B, Heidari M, Shekarkhar G. (2015). Gastrointestinal Basidiobolomycosis, a rare and under-diagnosed fungal infection in immunocompetent hosts: a review article. Iran J Med Sci.

